# [*O*-Ethyl *N*-(4-nitro­phen­yl)thio­carbam­ato-κ*S*](tri-*p*-tolyl­phosphine-κ*P*)gold(I)

**DOI:** 10.1107/S1600536808038257

**Published:** 2008-11-20

**Authors:** Grant A. Broker, Edward R. T. Tiekink

**Affiliations:** a Department of Chemistry, The University of Texas at San Antonio, One UTSA Circle, San Antonio, Texas 78249-0698, USA

## Abstract

A nearly linear coordination geometry for Au is found in the title compound, [Au(C_9_H_9_N_2_O_3_S)(C_21_H_21_P)]. The thio­carbamate ligand is orientated so that the aryl group is in close proximity to the Au atom, consistent with an Au⋯π contact [Au⋯*Cg* = 3.351 (5) Å; *Cg* is the centroid of the aromatic ring].

## Related literature

For related structures and discussion of structural diversity, see: Ho *et al.* (2006 ▶); Ho & Tiekink (2007 ▶); Kuan *et al.* (2008 ▶).
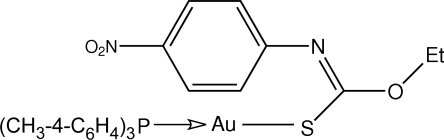


## Experimental

### 

#### Crystal data

[Au(C_9_H_9_N_2_O_3_S)(C_21_H_21_P)]
*M* *_r_* = 726.56
Monoclinic, 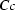

*a* = 16.622 (3) Å
*b* = 18.307 (4) Å
*c* = 10.094 (2) Å
β = 112.78 (3)°
*V* = 2832.0 (10) Å^3^
*Z* = 4
Mo *K*α radiation
μ = 5.36 mm^−1^
*T* = 173 (2) K
0.15 × 0.12 × 0.05 mm

#### Data collection

Rigaku AFC12K/SATURN724 diffractometer
Absorption correction: multi-scan (*ABSCOR*; Higashi, 1995 ▶) *T*_min_ = 0.739, *T*_max_ = 1.000 (expected range = 0.565–0.765)
9217 measured reflections
4917 independent reflections
4682 reflections with *I* > 2σ(*I*)
*R*_int_ = 0.059

#### Refinement

*R*[*F* ^2^ > 2σ(*F* ^2^)] = 0.048
*wR*(*F* ^2^) = 0.099
*S* = 1.06
4917 reflections
346 parameters
2 restraints
H-atom parameters constrained
Δρ_max_ = 1.37 e Å^−3^
Δρ_min_ = −2.40 e Å^−3^
Absolute structure: Flack (1983 ▶), 1980 Friedel pairs
Flack parameter: 0.008 (11)

### 

Data collection: *CrystalClear* (Rigaku/MSC, 2005 ▶); cell refinement: *CrystalClear*; data reduction: *CrystalClear*; program(s) used to solve structure: *SHELXS97* (Sheldrick, 2008 ▶); program(s) used to refine structure: *SHELXL97* (Sheldrick, 2008 ▶); molecular graphics: *ORTEPII* (Johnson, 1976 ▶); software used to prepare material for publication: *SHELXL97*.

## Supplementary Material

Crystal structure: contains datablocks global, I. DOI: 10.1107/S1600536808038257/ng2516sup1.cif

Structure factors: contains datablocks I. DOI: 10.1107/S1600536808038257/ng2516Isup2.hkl

Additional supplementary materials:  crystallographic information; 3D view; checkCIF report

## Figures and Tables

**Fig. 1.**
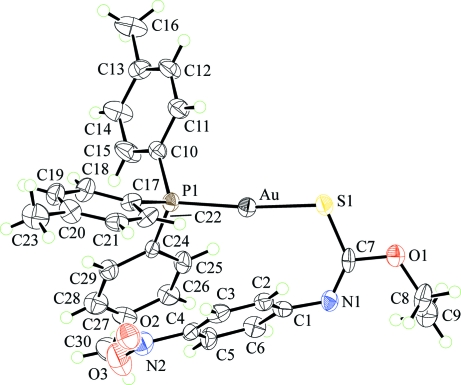
Molecular structure of (I) showing atom-labelling scheme and displacement ellipsoids at the 50% probability level.

## supplementary crystallographic information

### Comment

Phosphinegold(I) thiocarbamides uniformly adopt linear coordination geometries defined by a S and P donor set (Ho *et al.*, 2006; Ho & Tiekink, 2007; Kuan *et al.*, 2008). In these structures the thiocarbamide-O atom is normally located in close proximity to the Au atom but in cases where the donor ability of the phosphine ligand is increased, as in the structure of the title compound (I), a rotation about the S—C bond occurs and the N-bound aryl group is orientated towards the Au centre (Kuan *et al.*, 2008). In (I), Fig. 1, such a rotation has occurred so that the Au···*Cg* distance is 3.351 (5) Å. Interestingly, in the *O*-methyl derivative, the thiocarbamide molecule is situated to allow for an intramolecular Au···O contact (Kuan *et al.*, 2008) suggesting that replacing methyl with a more electronegative ethyl group is sufficient to introduce a difference in the orientation of the molecule.

### Experimental

The title compound (I) was prepared following established literature procedures (Ho *et al.*, 2006). Yellow crystals were obtained by the slow evaporation of an acetone solution of (I).

### Refinement

The H atoms were geometrically placed (C—H = 0.95–0.99 Å) and refined as riding with *U*_iso_(H) = 1.2*U*_eq_(C) or 1.5*U*_eq_(methyl C). The largest peak was 1.46 Å from Au and the deepest hole was 1.02 Å from Au.

### Crystal data

**Table:**

[Au(C_9_H_9_N_2_O_3_S)(C_21_H_21_P)]	*F*_000_ = 1432
*M**_r_* = 726.56	*D*_x_ = 1.704 Mg m^−^^3^
Monoclinic, *C**c*	Mo *K*α radiation λ = 0.71070 Å
Hall symbol: C -2yc	Cell parameters from 6817 reflections
*a* = 16.622 (3) Å	θ = 2.4–30.4º
*b* = 18.307 (4) Å	µ = 5.36 mm^−^^1^
*c* = 10.094 (2) Å	*T* = 173 (2) K
β = 112.78 (3)º	Prism, yellow
*V* = 2832.0 (10) Å^3^	0.15 × 0.12 × 0.05 mm
*Z* = 4	

### Data collection

**Table:**

Rigaku AFC12K/SATURN724 diffractometer	4917 independent reflections
Radiation source: fine-focus sealed tube	4682 reflections with *I* > 2σ(*I*)
Monochromator: graphite	*R*_int_ = 0.059
*T* = 173(2) K	θ_max_ = 26.5º
ω scans	θ_min_ = 2.4º
Absorption correction: multi-scan(ABSCOR; Higashi, 1995)	*h* = −20→20
*T*_min_ = 0.739, *T*_max_ = 1.000	*k* = −22→22
9217 measured reflections	*l* = −12→10

### Refinement

**Table:**

Refinement on *F*^2^	Hydrogen site location: inferred from neighbouring sites
Least-squares matrix: full	H-atom parameters constrained
*R*[*F*^2^ > 2σ(*F*^2^)] = 0.048	*w* = 1/[σ^2^(*F*_o_^2^) + (0.0306*P*)^2^ + 5.5648*P*] where *P* = (*F*_o_^2^ + 2*F*_c_^2^)/3
*wR*(*F*^2^) = 0.099	(Δ/σ)_max_ < 0.001
*S* = 1.06	Δρ_max_ = 1.37 e Å^−^^3^
4917 reflections	Δρ_min_ = −2.40 e Å^−^^3^
346 parameters	Extinction correction: none
2 restraints	Absolute structure: Flack (1983), 1980 Friedel pairs
Primary atom site location: structure-invariant direct methods	Flack parameter: 0.008 (11)
Secondary atom site location: difference Fourier map	

### Special details

**Table:**

Geometry. All e.s.d.'s (except the e.s.d. in the dihedral angle between two l.s. planes) are estimated using the full covariance matrix. The cell e.s.d.'s are taken into account individually in the estimation of e.s.d.'s in distances, angles and torsion angles; correlations between e.s.d.'s in cell parameters are only used when they are defined by crystal symmetry. An approximate (isotropic) treatment of cell e.s.d.'s is used for estimating e.s.d.'s involving l.s. planes.
Refinement. Refinement of *F*^2^ against ALL reflections. The weighted *R*-factor *wR* and goodness of fit *S* are based on *F*^2^, conventional *R*-factors *R* are based on *F*, with *F* set to zero for negative *F*^2^. The threshold expression of *F*^2^ > σ(*F*^2^) is used only for calculating *R*-factors(gt) *etc*. and is not relevant to the choice of reflections for refinement. *R*-factors based on *F*^2^ are statistically about twice as large as those based on *F*, and *R*- factors based on ALL data will be even larger.

### Fractional atomic coordinates and isotropic or equivalent isotropic displacement parameters (Å^2^)

**Table:**

	*x*	*y*	*z*	*U*_iso_*/*U*_eq_	
Au	0.49939 (4)	0.473591 (16)	0.74925 (5)	0.03122 (11)	
S1	0.59961 (18)	0.56583 (13)	0.8436 (3)	0.0367 (6)	
P1	0.39053 (16)	0.39085 (13)	0.6519 (3)	0.0270 (5)	
O1	0.7581 (5)	0.5885 (4)	0.8868 (7)	0.0355 (16)	
O2	0.5347 (7)	0.2127 (6)	1.0596 (12)	0.055 (3)	
O3	0.5424 (7)	0.1666 (5)	0.8655 (11)	0.074 (3)	
N1	0.7339 (6)	0.4654 (4)	0.8938 (9)	0.032 (2)	
N2	0.5563 (7)	0.2152 (6)	0.9524 (12)	0.049 (3)	
C1	0.6860 (6)	0.4052 (5)	0.9059 (12)	0.030 (2)	
C2	0.6591 (6)	0.3981 (6)	1.0185 (11)	0.031 (2)	
H2A	0.6693	0.4371	1.0851	0.037*	
C3	0.6179 (7)	0.3364 (6)	1.0369 (11)	0.035 (2)	
H3A	0.6012	0.3317	1.1166	0.042*	
C4	0.6014 (7)	0.2813 (6)	0.9367 (12)	0.031 (2)	
C5	0.6258 (7)	0.2864 (6)	0.8200 (12)	0.037 (2)	
H5A	0.6141	0.2479	0.7521	0.044*	
C6	0.6674 (7)	0.3490 (6)	0.8060 (11)	0.035 (2)	
H6A	0.6839	0.3540	0.7262	0.042*	
C7	0.7041 (7)	0.5312 (5)	0.8757 (11)	0.031 (2)	
C8	0.8498 (7)	0.5729 (7)	0.9210 (12)	0.046 (3)	
H8A	0.8685	0.5323	0.9913	0.055*	
H8B	0.8849	0.6165	0.9665	0.055*	
C9	0.8674 (9)	0.5524 (8)	0.7904 (14)	0.052 (3)	
H9A	0.9298	0.5423	0.8188	0.079*	
H9B	0.8504	0.5929	0.7214	0.079*	
H9C	0.8337	0.5088	0.7458	0.079*	
C10	0.2983 (7)	0.4272 (5)	0.5037 (11)	0.029 (2)	
C11	0.2704 (7)	0.4981 (6)	0.5072 (13)	0.041 (3)	
H11A	0.3001	0.5274	0.5895	0.050*	
C12	0.2010 (8)	0.5276 (5)	0.3950 (13)	0.042 (3)	
H12A	0.1827	0.5760	0.4029	0.050*	
C13	0.1572 (8)	0.4879 (7)	0.2704 (12)	0.038 (3)	
C14	0.1839 (9)	0.4159 (6)	0.2679 (12)	0.050 (3)	
H14A	0.1525	0.3860	0.1876	0.060*	
C15	0.2548 (9)	0.3868 (6)	0.3790 (13)	0.053 (3)	
H15A	0.2742	0.3389	0.3705	0.063*	
C16	0.0830 (10)	0.5201 (7)	0.1453 (14)	0.059 (4)	
H16A	0.0825	0.4991	0.0556	0.088*	
H16B	0.0902	0.5731	0.1441	0.088*	
H16C	0.0277	0.5088	0.1545	0.088*	
C17	0.3478 (6)	0.3540 (5)	0.7770 (10)	0.028 (2)	
C18	0.2710 (7)	0.3149 (6)	0.7351 (11)	0.038 (2)	
H18A	0.2356	0.3105	0.6357	0.045*	
C19	0.2448 (7)	0.2826 (6)	0.8332 (12)	0.038 (2)	
H19A	0.1913	0.2565	0.8008	0.045*	
C20	0.2947 (8)	0.2870 (6)	0.9794 (11)	0.039 (3)	
C21	0.3698 (7)	0.3280 (6)	1.0242 (11)	0.036 (2)	
H21A	0.4037	0.3329	1.1241	0.043*	
C22	0.3968 (6)	0.3624 (6)	0.9264 (11)	0.037 (2)	
H22A	0.4481	0.3915	0.9593	0.044*	
C23	0.2631 (9)	0.2489 (7)	1.0866 (13)	0.054 (3)	
H23A	0.3121	0.2438	1.1796	0.081*	
H23B	0.2402	0.2004	1.0500	0.081*	
H23C	0.2170	0.2782	1.0985	0.081*	
C24	0.4231 (6)	0.3135 (5)	0.5721 (10)	0.025 (2)	
C25	0.4745 (7)	0.3277 (6)	0.4931 (11)	0.037 (2)	
H25A	0.4953	0.3758	0.4900	0.044*	
C26	0.4948 (7)	0.2714 (6)	0.4193 (11)	0.036 (2)	
H26A	0.5285	0.2818	0.3642	0.043*	
C27	0.4667 (10)	0.1993 (7)	0.4240 (15)	0.041 (3)	
C28	0.4187 (8)	0.1867 (7)	0.5063 (14)	0.044 (3)	
H28A	0.4002	0.1383	0.5129	0.053*	
C29	0.3960 (7)	0.2420 (6)	0.5805 (12)	0.036 (2)	
H29A	0.3626	0.2313	0.6360	0.043*	
C30	0.4884 (10)	0.1410 (7)	0.3408 (14)	0.058 (3)	
H30A	0.4559	0.0965	0.3420	0.087*	
H30B	0.5511	0.1308	0.3842	0.087*	
H30C	0.4725	0.1572	0.2413	0.087*	

### Atomic displacement parameters (Å^2^)

**Table:**

	*U*^11^	*U*^22^	*U*^33^	*U*^12^	*U*^13^	*U*^23^
Au	0.03049 (18)	0.03164 (17)	0.03174 (19)	−0.0017 (2)	0.01226 (14)	0.0001 (2)
S1	0.0368 (14)	0.0270 (11)	0.0499 (16)	−0.0055 (11)	0.0207 (13)	−0.0056 (11)
P1	0.0262 (13)	0.0295 (12)	0.0239 (13)	−0.0029 (10)	0.0083 (11)	0.0002 (10)
O1	0.036 (4)	0.036 (4)	0.035 (4)	−0.005 (3)	0.015 (3)	0.000 (3)
O2	0.059 (7)	0.052 (6)	0.061 (7)	−0.001 (5)	0.032 (6)	0.022 (5)
O3	0.091 (8)	0.051 (5)	0.070 (7)	−0.019 (6)	0.021 (6)	0.001 (5)
N1	0.030 (5)	0.031 (4)	0.030 (5)	−0.007 (4)	0.006 (4)	−0.007 (4)
N2	0.037 (6)	0.047 (6)	0.042 (7)	−0.006 (5)	−0.007 (5)	0.008 (5)
C1	0.029 (5)	0.028 (5)	0.032 (6)	0.007 (4)	0.011 (5)	0.004 (4)
C2	0.029 (6)	0.033 (5)	0.030 (6)	0.003 (4)	0.012 (5)	0.003 (4)
C3	0.033 (6)	0.048 (6)	0.022 (5)	0.009 (5)	0.008 (5)	0.010 (5)
C4	0.021 (5)	0.035 (6)	0.035 (6)	0.001 (5)	0.009 (5)	0.004 (5)
C5	0.033 (6)	0.032 (5)	0.047 (7)	−0.010 (5)	0.018 (5)	−0.013 (5)
C6	0.044 (6)	0.040 (6)	0.027 (6)	0.003 (5)	0.019 (5)	−0.002 (5)
C7	0.033 (6)	0.035 (5)	0.025 (5)	−0.018 (4)	0.012 (5)	−0.001 (4)
C8	0.036 (6)	0.060 (7)	0.036 (6)	−0.020 (6)	0.008 (5)	−0.002 (5)
C9	0.049 (8)	0.056 (8)	0.049 (8)	−0.011 (7)	0.015 (7)	−0.002 (7)
C10	0.034 (6)	0.027 (5)	0.029 (6)	0.000 (4)	0.017 (5)	0.002 (4)
C11	0.042 (7)	0.030 (5)	0.046 (7)	0.002 (5)	0.010 (6)	−0.002 (5)
C12	0.049 (7)	0.024 (5)	0.042 (7)	0.003 (5)	0.006 (6)	−0.003 (4)
C13	0.036 (6)	0.046 (6)	0.031 (6)	0.007 (5)	0.013 (5)	0.011 (5)
C14	0.068 (9)	0.041 (6)	0.026 (6)	0.016 (6)	0.001 (6)	−0.006 (5)
C15	0.067 (9)	0.028 (5)	0.049 (7)	0.007 (6)	0.008 (7)	−0.008 (5)
C16	0.077 (10)	0.050 (7)	0.046 (8)	0.028 (7)	0.021 (8)	0.009 (6)
C17	0.030 (5)	0.039 (5)	0.021 (5)	0.007 (4)	0.016 (5)	0.010 (4)
C18	0.033 (6)	0.052 (6)	0.025 (5)	−0.008 (5)	0.009 (5)	−0.001 (5)
C19	0.031 (6)	0.043 (6)	0.038 (6)	−0.006 (5)	0.012 (5)	0.005 (5)
C20	0.052 (7)	0.041 (6)	0.024 (6)	−0.003 (5)	0.014 (5)	−0.001 (5)
C21	0.040 (6)	0.036 (5)	0.032 (6)	0.009 (5)	0.013 (5)	−0.004 (4)
C22	0.024 (5)	0.051 (6)	0.033 (6)	0.002 (5)	0.009 (5)	0.000 (5)
C23	0.068 (9)	0.059 (8)	0.043 (7)	0.006 (7)	0.032 (7)	0.014 (6)
C24	0.019 (5)	0.029 (5)	0.024 (5)	−0.005 (4)	0.004 (4)	0.005 (4)
C25	0.038 (6)	0.036 (5)	0.044 (6)	−0.006 (5)	0.025 (6)	0.006 (5)
C26	0.044 (6)	0.039 (6)	0.036 (6)	0.005 (5)	0.029 (5)	0.002 (5)
C27	0.047 (8)	0.035 (6)	0.039 (8)	0.013 (6)	0.015 (7)	0.008 (5)
C28	0.049 (8)	0.048 (7)	0.044 (8)	0.005 (6)	0.025 (7)	0.002 (6)
C29	0.036 (6)	0.036 (6)	0.037 (6)	−0.001 (5)	0.015 (5)	0.005 (5)
C30	0.080 (10)	0.049 (7)	0.051 (8)	0.010 (7)	0.032 (8)	−0.003 (6)

### Geometric parameters (Å, °)

**Table:**

Au—P1	2.271 (3)	C13—C16	1.502 (17)
Au—S1	2.303 (3)	C14—C15	1.380 (17)
S1—C7	1.757 (11)	C14—H14A	0.9500
P1—C17	1.801 (9)	C15—H15A	0.9500
P1—C10	1.804 (11)	C16—H16A	0.9800
P1—C24	1.813 (10)	C16—H16B	0.9800
O1—C7	1.358 (11)	C16—H16C	0.9800
O1—C8	1.454 (13)	C17—C18	1.380 (14)
O2—N2	1.266 (15)	C17—C22	1.417 (14)
O3—N2	1.207 (14)	C18—C19	1.361 (13)
N1—C7	1.287 (12)	C18—H18A	0.9500
N1—C1	1.392 (12)	C19—C20	1.388 (15)
N2—C4	1.464 (15)	C19—H19A	0.9500
C1—C2	1.380 (13)	C20—C21	1.375 (16)
C1—C6	1.389 (14)	C20—C23	1.540 (14)
C2—C3	1.371 (15)	C21—C22	1.384 (14)
C2—H2A	0.9500	C21—H21A	0.9500
C3—C4	1.378 (16)	C22—H22A	0.9500
C3—H3A	0.9500	C23—H23A	0.9800
C4—C5	1.390 (14)	C23—H23B	0.9800
C5—C6	1.374 (14)	C23—H23C	0.9800
C5—H5A	0.9500	C24—C29	1.397 (13)
C6—H6A	0.9500	C24—C25	1.400 (12)
C8—C9	1.505 (16)	C25—C26	1.388 (14)
C8—H8A	0.9900	C25—H25A	0.9500
C8—H8B	0.9900	C26—C27	1.407 (17)
C9—H9A	0.9800	C26—H26A	0.9500
C9—H9B	0.9800	C27—C28	1.376 (18)
C9—H9C	0.9800	C27—C30	1.487 (17)
C10—C11	1.384 (14)	C28—C29	1.396 (16)
C10—C15	1.396 (15)	C28—H28A	0.9500
C11—C12	1.376 (16)	C29—H29A	0.9500
C11—H11A	0.9500	C30—H30A	0.9800
C12—C13	1.389 (16)	C30—H30B	0.9800
C12—H12A	0.9500	C30—H30C	0.9800
C13—C14	1.395 (15)		
			
P1—Au—S1	174.54 (10)	C15—C14—H14A	119.1
C7—S1—Au	108.4 (3)	C13—C14—H14A	119.1
C17—P1—C10	106.3 (5)	C14—C15—C10	120.6 (10)
C17—P1—C24	106.6 (4)	C14—C15—H15A	119.7
C10—P1—C24	102.9 (4)	C10—C15—H15A	119.7
C17—P1—Au	114.2 (4)	C13—C16—H16A	109.5
C10—P1—Au	113.1 (3)	C13—C16—H16B	109.5
C24—P1—Au	112.8 (3)	H16A—C16—H16B	109.5
C7—O1—C8	117.8 (8)	C13—C16—H16C	109.5
C7—N1—C1	123.4 (9)	H16A—C16—H16C	109.5
O3—N2—O2	123.8 (11)	H16B—C16—H16C	109.5
O3—N2—C4	119.5 (11)	C18—C17—C22	117.6 (8)
O2—N2—C4	116.7 (11)	C18—C17—P1	123.3 (8)
C2—C1—C6	118.4 (9)	C22—C17—P1	119.0 (8)
C2—C1—N1	121.7 (10)	C19—C18—C17	121.4 (10)
C6—C1—N1	119.8 (9)	C19—C18—H18A	119.3
C3—C2—C1	121.9 (10)	C17—C18—H18A	119.3
C3—C2—H2A	119.1	C18—C19—C20	121.4 (10)
C1—C2—H2A	119.1	C18—C19—H19A	119.3
C2—C3—C4	118.1 (9)	C20—C19—H19A	119.3
C2—C3—H3A	120.9	C21—C20—C19	118.3 (10)
C4—C3—H3A	120.9	C21—C20—C23	122.0 (10)
C3—C4—C5	122.1 (10)	C19—C20—C23	119.7 (11)
C3—C4—N2	119.4 (10)	C20—C21—C22	121.2 (10)
C5—C4—N2	118.5 (10)	C20—C21—H21A	119.4
C6—C5—C4	117.9 (10)	C22—C21—H21A	119.4
C6—C5—H5A	121.0	C21—C22—C17	119.9 (10)
C4—C5—H5A	121.0	C21—C22—H22A	120.0
C5—C6—C1	121.5 (9)	C17—C22—H22A	120.0
C5—C6—H6A	119.3	C20—C23—H23A	109.5
C1—C6—H6A	119.3	C20—C23—H23B	109.5
N1—C7—O1	120.3 (10)	H23A—C23—H23B	109.5
N1—C7—S1	131.5 (8)	C20—C23—H23C	109.5
O1—C7—S1	108.1 (7)	H23A—C23—H23C	109.5
O1—C8—C9	112.5 (9)	H23B—C23—H23C	109.5
O1—C8—H8A	109.1	C29—C24—C25	119.5 (9)
C9—C8—H8A	109.1	C29—C24—P1	123.0 (8)
O1—C8—H8B	109.1	C25—C24—P1	117.4 (7)
C9—C8—H8B	109.1	C26—C25—C24	119.7 (9)
H8A—C8—H8B	107.8	C26—C25—H25A	120.1
C8—C9—H9A	109.5	C24—C25—H25A	120.1
C8—C9—H9B	109.5	C25—C26—C27	121.7 (9)
H9A—C9—H9B	109.5	C25—C26—H26A	119.2
C8—C9—H9C	109.5	C27—C26—H26A	119.2
H9A—C9—H9C	109.5	C28—C27—C26	117.1 (12)
H9B—C9—H9C	109.5	C28—C27—C30	123.1 (13)
C11—C10—C15	117.3 (10)	C26—C27—C30	119.9 (11)
C11—C10—P1	120.7 (8)	C27—C28—C29	122.9 (12)
C15—C10—P1	122.0 (8)	C27—C28—H28A	118.5
C12—C11—C10	121.9 (10)	C29—C28—H28A	118.5
C12—C11—H11A	119.0	C24—C29—C28	119.0 (10)
C10—C11—H11A	119.0	C24—C29—H29A	120.5
C11—C12—C13	121.3 (10)	C28—C29—H29A	120.5
C11—C12—H12A	119.3	C27—C30—H30A	109.5
C13—C12—H12A	119.3	C27—C30—H30B	109.5
C12—C13—C14	116.8 (10)	H30A—C30—H30B	109.5
C12—C13—C16	121.9 (11)	C27—C30—H30C	109.5
C14—C13—C16	121.3 (12)	H30A—C30—H30C	109.5
C15—C14—C13	121.9 (11)	H30B—C30—H30C	109.5
			
P1—Au—S1—C7	−170.4 (10)	C12—C13—C14—C15	−4.9 (19)
S1—Au—P1—C17	−86.1 (11)	C16—C13—C14—C15	176.6 (12)
S1—Au—P1—C10	35.6 (12)	C13—C14—C15—C10	5(2)
S1—Au—P1—C24	151.9 (10)	C11—C10—C15—C14	−3.5 (18)
C7—N1—C1—C2	−63.7 (14)	P1—C10—C15—C14	179.4 (10)
C7—N1—C1—C6	119.0 (11)	C10—P1—C17—C18	41.1 (10)
C6—C1—C2—C3	2.6 (15)	C24—P1—C17—C18	−68.2 (9)
N1—C1—C2—C3	−174.8 (9)	Au—P1—C17—C18	166.5 (8)
C1—C2—C3—C4	−1.9 (15)	C10—P1—C17—C22	−141.8 (8)
C2—C3—C4—C5	0.6 (16)	C24—P1—C17—C22	108.9 (9)
C2—C3—C4—N2	−179.0 (9)	Au—P1—C17—C22	−16.5 (9)
O3—N2—C4—C3	−179.4 (11)	C22—C17—C18—C19	−3.0 (15)
O2—N2—C4—C3	0.1 (16)	P1—C17—C18—C19	174.2 (9)
O3—N2—C4—C5	0.9 (16)	C17—C18—C19—C20	−0.6 (17)
O2—N2—C4—C5	−179.6 (10)	C18—C19—C20—C21	3.2 (16)
C3—C4—C5—C6	−0.2 (16)	C18—C19—C20—C23	−179.4 (10)
N2—C4—C5—C6	179.5 (10)	C19—C20—C21—C22	−2.0 (16)
C4—C5—C6—C1	1.0 (17)	C23—C20—C21—C22	−179.4 (10)
C2—C1—C6—C5	−2.1 (16)	C20—C21—C22—C17	−1.5 (15)
N1—C1—C6—C5	175.3 (10)	C18—C17—C22—C21	4.0 (15)
C1—N1—C7—O1	169.9 (9)	P1—C17—C22—C21	−173.2 (8)
C1—N1—C7—S1	−7.1 (16)	C17—P1—C24—C29	15.8 (10)
C8—O1—C7—N1	−0.7 (13)	C10—P1—C24—C29	−95.9 (9)
C8—O1—C7—S1	176.9 (7)	Au—P1—C24—C29	142.0 (8)
Au—S1—C7—N1	−21.9 (11)	C17—P1—C24—C25	−167.3 (8)
Au—S1—C7—O1	160.9 (5)	C10—P1—C24—C25	81.1 (9)
C7—O1—C8—C9	83.3 (12)	Au—P1—C24—C25	−41.1 (9)
C17—P1—C10—C11	87.5 (9)	C29—C24—C25—C26	2.7 (16)
C24—P1—C10—C11	−160.6 (8)	P1—C24—C25—C26	−174.4 (9)
Au—P1—C10—C11	−38.6 (9)	C24—C25—C26—C27	−1.4 (18)
C17—P1—C10—C15	−95.5 (10)	C25—C26—C27—C28	−1(2)
C24—P1—C10—C15	16.3 (10)	C25—C26—C27—C30	178.7 (12)
Au—P1—C10—C15	138.4 (9)	C26—C27—C28—C29	1(2)
C15—C10—C11—C12	2.0 (17)	C30—C27—C28—C29	−177.9 (13)
P1—C10—C11—C12	179.2 (9)	C25—C24—C29—C28	−1.9 (16)
C10—C11—C12—C13	−2.1 (18)	P1—C24—C29—C28	175.0 (9)
C11—C12—C13—C14	3.3 (18)	C27—C28—C29—C24	−0.3 (19)
C11—C12—C13—C16	−178.2 (11)		
